# Planned early delivery for late preterm pre-eclampsia in a low- and middle-income setting: a feasibility study

**DOI:** 10.1186/s12978-021-01159-y

**Published:** 2021-06-02

**Authors:** Alice Beardmore-Gray, Nicola Vousden, Sergio A. Silverio, Umesh Charantimath, Geetanjali Katageri, Mrutyunjaya Bellad, Sebastian Chinkoyo, Bellington Vwalika, Shivaprasad Goudar, Jane Sandall, Lucy C. Chappell, Andrew H. Shennan

**Affiliations:** 1 Department of Women and Children’s Health, School of Life Course Sciences, King’s College London, London, UK; 2 Women’s and Children’s Health Research Unit, KLE Academy of Higher Education and Research, JNMC, Belagavi, Karnataka India; 3 BVV Sangha’s S Nijalingappa Medical College and HSK Hospital and Research Centre, Bagalkot, Karnataka India; 4 Department of Obstetrics and Gynaecology, Ndola Teaching Hospital, Ndola, Zambia; 5 Department of Obstetrics and Gynaecology, University of Zambia, Lusaka, Zambia

**Keywords:** Pregnancy, Pre-eclampsia, Delivery, Low- and middle-income, Feasibility, Acceptability

## Abstract

**Background:**

Pre-eclampsia is a leading cause of maternal and perinatal mortality and morbidity globally. Planned delivery between 34^+0^ and 36^+6^ weeks may reduce adverse pregnancy outcomes but is yet to be evaluated in a low and middle-income setting. Prior to designing a randomised controlled trial to evaluate this in India and Zambia, we carried out a 6-month feasibility study in order to better understand the proposed trial environment and guide development of our intervention.

**Methods:**

We used mixed methods to understand the disease burden and current management of pre-eclampsia at our proposed trial sites and explore the acceptability of the intervention. We undertook a case notes review of women with pre-eclampsia who delivered at the proposed trial sites over a 3-month period, alongside facilitating focus group discussions with women and partners and conducting semi-structured interviews with healthcare providers. Descriptive statistics were used to analyse audit data. A thematic framework analysis was used for qualitative data.

**Results:**

Case notes data (n = 326) showed that in our settings, 19.5% (n = 44) of women with pre-eclampsia delivering beyond 34 weeks experienced an adverse outcome. In women delivering between 34^+0^ and 36^+6^ weeks, there were similar numbers of antenatal stillbirths [n = 3 (3.3%)] and neonatal deaths [n = 3 (3.4%)]; median infant birthweight was 2.2 kg and 1.9 kg in Zambia and India respectively. Lived experience of women and healthcare providers was an important facilitator to the proposed intervention, highlighting the serious consequences of pre-eclampsia. A preference for spontaneous labour and limited neonatal resources were identified as potential barriers.

**Conclusions:**

This study demonstrated a clear need to evaluate the intervention and highlighted several challenges relating to trial context that enabled us to adapt our protocol and design an acceptable intervention. Our study demonstrates the importance of assessing feasibility when developing complex interventions, particularly in a low-resource setting. Additionally, it provides a unique insight into the management of pre-eclampsia at our trial settings and an understanding of the knowledge, attitudes and beliefs underpinning the acceptability of planned early delivery.

**Supplementary Information:**

The online version contains supplementary material available at 10.1186/s12978-021-01159-y.

## Plain language summary

Pre-eclampsia is a complication of pregnancy and is one of the major causes of pregnancy-related death and serious illness for women and babies around the world. Most of these deaths occur in lower income countries in Africa and Asia. Signs of pre-eclampsia include high blood pressure and protein in the urine. It is unpredictable and may affect different organs within the woman, leading to seizures, stroke and even death if not well managed. It can also affect the baby’s growth and in severe cases lead to stillbirth. We know that birth of the baby (and placenta) is the only cure for pre-eclampsia. Currently, it is recommended by the World Health Organisation that all women with pre-eclampsia are offered planned early birth once they reach 37 weeks of pregnancy, unless they develop severe complications needing intervention sooner than this. However, research from higher income countries has shown that planned early birth from 34 weeks of pregnancy may reduce serious complications in the woman, without causing harm to the baby. We are designing a clinical trial to find out whether, in women with pre-eclampsia between 34 and 37 weeks of pregnancy, it is better to offer planned early birth or to offer close monitoring until either they reach 37 weeks, or a complication develops requiring emergency intervention. Before designing this trial, we carried out a study in order to establish whether the main trial would be possible, and acceptable to the local community, at our potential trial sites in India and Zambia.

**Supplementary Information:**

The online version contains supplementary material available at 10.1186/s12978-021-01159-y.

## Background

The disproportionate burden of pre-eclampsia in low and middle-income countries (LMIC), particularly in Sub-Saharan Africa and South Asia, is well described [1–3]. Hypertensive disorders are the second biggest cause of maternal mortality worldwide [2], and pre-eclampsia itself is responsible for 76,000 maternal deaths and 500,000 perinatal deaths every year [4].The vast majority of these (98%) occur in LMIC [1]. Despite this, there is a lack of research into interventions which could be implemented in these regions in order to improve pregnancy outcomes. One such intervention, planned early delivery, has been shown to reduce adverse maternal outcomes in a high-income setting [5, 6], but is yet to be evaluated in a LMIC setting. The proposed CRADLE-4 trial aims to establish whether planned early delivery in women with late preterm pre-eclampsia (between 34^+0^- and 36^+6^- weeks’ gestation) is effective in reducing adverse pregnancy outcomes in India and Zambia. To our knowledge, it will be the first trial to evaluate timing of delivery in late preterm pre-eclampsia in LMIC. It is now widely recognised that conducting an assessment of feasibility is an essential step prior to the development and evaluation of a healthcare intervention as part of a larger-scale clinical trial [7, 8]. We therefore designed this initial feasibility study in order to understand the contextual factors likely to influence trial implementation and assess the perceived barriers and facilitators to the intervention. The findings were used to directly inform the design of the main trial protocol. We anticipate that the results of this study would not just optimise delivery of the trial itself, but also improve the external validity of any significant trial findings such that they are generalisable to similar settings and practicable to implement in a real-world environment.

## Methods

### Aims and objectives

The overall aim of this study was to explore the feasibility of planned early delivery in women with pre-eclampsia (not requiring immediate delivery) between 34^+0^- and 36^+6^-weeks’gestation in order to inform the design of the intervention and the main trial protocol. By assessing feasibility, we aimed to explore areas of uncertainty surrounding the main trial design. Specific study objectives were to confirm the need for the proposed intervention, obtain estimates to help with sample size calculation, explore potential outcome measures, understand the resource limitations likely to impact upon overall study design and to establish whether the proposed intervention would be acceptable to all stakeholders (pregnant women, their partners and relevant healthcare providers). In order to meet these objectives we set out to understand the disease burden associated with pre-eclampsia at the proposed trial sites, understand the current management of pregnancies complicated by pre-eclampsia at the proposed trial sites, and to explore the perceived risks and benefits of the intervention by women, their partners and healthcare providers involved in the delivery of maternal and new-born healthcare.

Ethical approval was provided by King’s College London Research Ethics Committee (LRS-18/19-8818), University of Zambia Research Ethics Committee (014-11-18) and KLES Academy of Higher Education and Research Institutional Ethics Committee (KAHER/IEC/2019-20/D-2742).

### Study design

CRADLE-4 Phase 1 study was designed as a mixed-methods [9] feasibility study which took place over a six-month period from 1st January 2019 to 30th June 2019. We chose to include qualitative research methods, which have gained increasing recognition for their important contribution to feasibility studies [10] and may be the most effective way of exploring key areas of uncertainty such as acceptability and local context. They are also increasingly used to address important questions about health and healthcare, particularly relevant in fields such as women’s health where, for example, understanding women’s experiences of childbirth is critical to the delivery of respectful maternity care [11]. In this study, we used a parallel approach [12], whereby quantitative and qualitative data collection and analysis were conducted separately and simultaneously and brought together at the interpretation stage [13]. This is a pragmatic approach to integration for such datasets [14] and allowed for qualitative data to complement and explain interesting findings from the quantitative data analysis. Analysis and interpretation of these integrated data was therefore exploratory, reflecting guidance for mixed methods feasibility studies [10].

### Study settings

The study was conducted across four of the proposed sites for the interventional phase of the trial in India and Zambia. These are tertiary level hospitals (providing Comprehensive Emergency Obstetric and Newborn Care) situated in urban environments:

- University Teaching Hospital, Lusaka, Zambia
- Ndola Teaching Hospital, Ndola, Zambia
- KLE Academy of Higher Education and Research’s, J N Medical College Hospital, Belgaum, Karnataka, India
- S Nijalingappa Medical College and Hanagal Shri Kumareshwar Hospital and Research Centre, Bagalkot, Karnataka, India

An additional site, Chipata first level hospital, was also used to facilitate two of the focus group discussions in Lusaka, Zambia.

### Case notes review

We undertook a retrospective case notes review of all women with pre-eclampsia who delivered at the study sites between January and March 2019. Following discussion with local site teams and initial site visits, and noting the high prevalence of pre-eclampsia and maternal morbidity in these settings, a three month period was deemed adequate to provide a reliable estimate of the number of women who would be potentially eligible for the main trial. A retrospective assessment of pre-eclampsia cases at these facilities over the preceding year did not indicate any meaningful seasonal variation that might influence these results. We also collected key maternal and infant outcomes to inform selection of primary and secondary outcomes and undertake a power calculation for the main trial. Women’s data were included if they had been diagnosed with pre-eclampsia and delivered at one of the participating sites. Relevant clinical notes were identified using ward registers with a record of diagnosis (e.g., pre-eclampsia) at discharge. The corresponding neonatal files were then located in order to record neonatal outcomes. Data were collected directly from case records by trained research assistants at each site. Study data were collected and managed using Research Electronic Data Capture Tools (REDCap). Whilst every effort was made to directly enter data onto REDCap, where internet connectivity made this impossible, data were entered onto paper case report forms (CRFs) and then inputted onto REDCap. Information was collected on baseline demographics, current pregnancy details, methods of gestational age determination, use of pre-eclampsia diagnostic criteria, clinical management of pre-eclampsia and gestation specific maternal and neonatal outcomes.

### Focus group discussions

In order to assess acceptability of the intervention to women and their families, we facilitated separate focus group discussions for pregnant women and their male partners (or closest supporting relative such as mother or mother-in-law). In both India and Zambia, women are generally considered to have low-decision making power in their households, particularly in relation to decisions on healthcare and how to use cash earnings[15, 15]. We therefore identified male partners as being an important group to include in the feasibility study, recognising they may exert considerable influence over a woman’s choice whether to participate in a research study or not. Participants were considered eligible if either they or their partner (or relative) were attending for routine antenatal care at any of the study sites. Individuals invited to take part were provided with written information detailing what their participation would involve (approximately one hour of audio-recorded focus group discussion) and written informed consent was obtained from all participants prior to initiation of the focus group discussion. Each focus group discussion was facilitated by a member of the local research team with previous experience in qualitative health research, using the local language preferred by participants (either Nyanja or Bemba in Zambia, or Kannada in India). Discussions took place in private spaces within the healthcare facility (e.g., seminar room). Refreshments were provided and transport costs were reimbursed. A focus group discussion guide (Additional file 1) was used to explore key questions relating to participants’ knowledge of pre-eclampsia, attitudes and beliefs towards planned early delivery and previous lived experience of hypertensive disorders of pregnancy. Each discussion was audio recorded, transcribed, translated, and subsequently analysed using NVivo qualitative data analysis software.

### Key stakeholder interviews

Semi-structured interviews were used to explore the acceptability of the intervention to healthcare providers. A stratified, purposive, sampling strategy [17] was used to identify key stakeholders, with individuals selected based on their potential influence in the main trial, following discussion with each of the local site teams. We identified a cross-section of staff involved in the delivery of maternal and newborn care across study sites which included obstetricians, paediatricians, midwives, maternity nurses and neonatal nurses. These individuals were then invited (either by phone, e-mail, or in person) to take part in a semi-structured interview, lasting approximately 30 min. Following an invitation to participate, each individual was provided with written information about what their participation would involve, and if willing to take part they were asked to provide written informed consent. Interviews were conducted at times convenient for the participant and private office spaces were used. A topic guide (Additional file 2) was used to explore participants’ understanding of pre-eclampsia, their clinical experience of the condition and the perceived risks and benefits of planned early delivery between 34^+0^- and 36^+6^-weeks’ gestation in women with pre-eclampsia. The interviews were conducted in English (as this was the professional working language at each of the study sites), and discussions were audio recorded, transcribed, and subsequently analysed using NVivo qualitative data analysis software.

### Data analysis

Descriptive analysis and summary statistics were used for the quantitative data generated from the case notes review. Qualitative data generated from the focus group discussions and stakeholder interviews were initially analysed separately and then combined. Triangulation of qualitative data (i.e., combining data from interviews and focus groups) in this way has been shown to enhance understanding of complex phenomena [13, 13]. Data were analysed using a thematic framework analysis appropriate to cross-disciplinary health research [18]. This adopts a deductive approach which enabled themes to be developed based on a combination of a priori research questions [19]. Thematic framework analysis is used to show presence and absence of patterns amongst different groups and does not rely on data saturation. Nevertheless, we adopted a pragmatic approach to data collection, continuing until we were satisfied enough data had been collected covering all major themes in the framework.

**Fig. 1:**
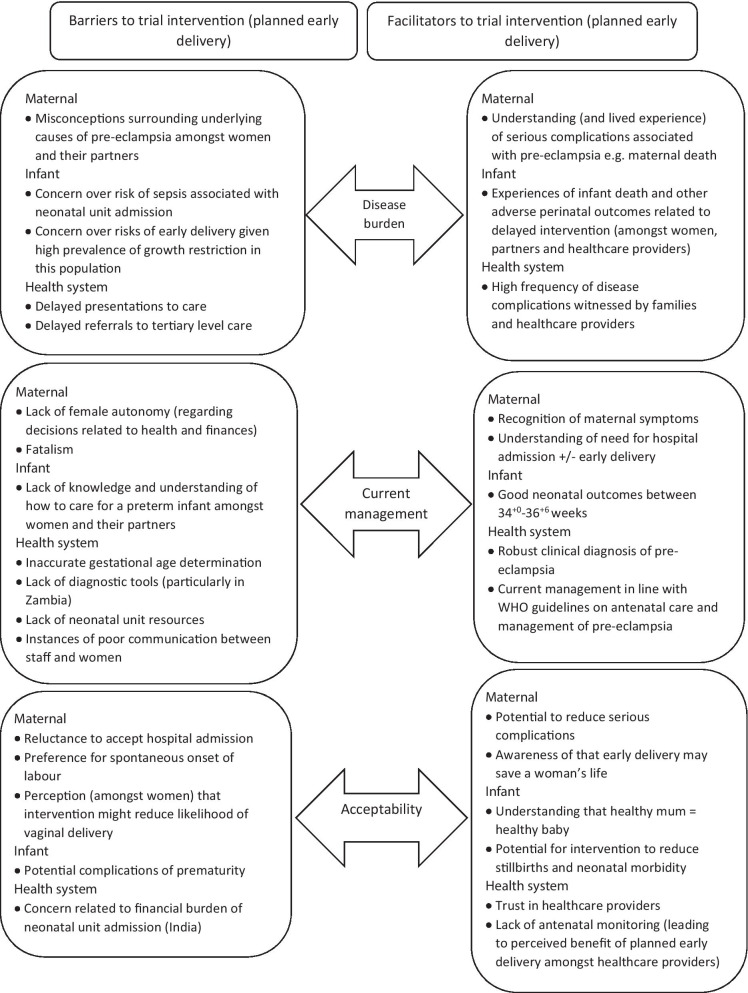
Integrated summary of key themes and findings

The thematic framework (Fig. 1) assessed three key domains, reflecting the study objectives: understanding disease burden of pre-eclampsia; current management of pre-eclampsia; and the acceptability of planned early delivery. Each of these were evaluated from a maternal perspective, an infant perspective, and a health system perspective.

The domains of disease burden and current management were chosen in order to explore the need for the intervention and understand the contextual factors likely to impact trial implementation. They were also considered to be important determinants of acceptability as they may influence the perceived risks and benefits that women and healthcare providers attribute to the intervention as a result of their experiences. Understanding these perceptions at an early stage of trial development was seen as an important step, not just in assessing the feasibility of the trial itself, but also the long-term feasibility of the intervention, should the main trial prove it to be effective.

## Results

Medical records for 326 women with pre-eclampsia (and 342 infants) who delivered at one of the study sites between January and March 2019 were included in the case notes review. A total of eight focus group discussions (n = 59 participants) took place with the number of participants in each focus group ranging between six and ten. Five focus group discussions involved pregnant women attending for routine antenatal care (four in Zambia, n = 29 participants; one in India, n = 6 participants) and three separate focus groups were facilitated with their male partners (two in Zambia, n = 17 participants; one in India, n = 7 participants). A total of 29 healthcare providers were interviewed. This purposive sample included nine obstetricians (Zambia n = 6 , India n = 3), six paediatricians (Zambia n = 2, India n = 4), six midwives (Zambia n = 6), two maternity nurses (India n = 2), five neonatal nurses (Zambia n = 3, India n = 2), and one healthcare assistant (India n = 1). An integrated summary of key qualitative and quantitative findings, presented according to the thematic framework, is shown below in Fig. 1. Key maternal data are shown in Table 1 and infant data in Table 2, grouped by gestational age (34^+0^–36^+6^ and ≥ 37 weeks). Illustrative quotes drawn from qualitative data are found in Table 3. Supplementary case notes review data are presented in Additional file 3 (Tables 4, 5).

**Table 1:** Case notes review—maternal data

	**34–36**^**+6**^** weeks N (%)**		** ≥ 37 weeks N (%)**	
	Zambian sites	Indian sites	Zambian sites	Indian sites
Total number of women	n = 69	n = 15	n = 98	n = 44
Maternal characteristics				
Mean (SD) age (years)	26.5 (7.0)	24.5 (3.2)	25.8 (5.9)	24.4. (4.2)
Primiparous	28 (40.5)	10 (66.7)	57 (58.2)	31 (70.5)
Singleton pregnancy	64 (92.8)	14 (93.3)	94 (95.9)	44 (100)
Ultrasound scan during pregnancy	44 (63.8)	8 (53.3)*	63 (64.3)	33 (75.0)*
At pre-eclampsia diagnosis				
SBP ≥ 140 or DBP ≥ 90 mmHg	68 (98.6)	11 (73.3)*	93 (94.9)	30 (68.2)*
≥ 1 + protein on urine dipstick	62 (89.9)	8 (53.3)	83 (84.7)	21 (47.7)
Quantitative assessment of proteinuria	0	0	0	0
Creatinine tested	18 (26.1)	15 (100)	23 (23.5)	42 (95.5)
Liver enzymes tested	24 (34.8)	15 (100)	24 (24.5)	42 (95.5)
Platelets tested	49 (71.0)	15 (100)	60 (61.2)	41 (93.2)
Pre-eclampsia management				
Given antihypertensives	61 (88.4)	15 (100)	88 (89.8)	35 (79.5)
> 1 antihypertensive agent	56 (81.6)	8 (53.3)	70 (71.4)	14 (31.8)
Received antenatal corticosteroids	42 (60.9)	4 (26.7)	9 (9.2)	1 (2.3)
Received magnesium sulfate	47 (68.1)	12 (80.0)	61 (62.2)	19 (43.2)
Admitted antenatally	66 (95.7)	15 (100)	90 (91.8)	44 (100)
Onset of labour:				
Spontaneous	22 (31.9)	3 (20.0)	43 (43.9)	24 (54.5)
Induced	25 (34.8)	4 (26.7)	28 (28.6)	5 (11.4)
Pre-labour caesarean section	22 (31.9)	8 (53.3)	27 (27.6)	15 (34.1)
Not documented	0	0	0	0
Composite of severe maternal mortality and morbidity (N women)	12 (17.4)	6 (40.0)	17 (17.3)	9 (20.5)
Individual components (non-exclusive events):				
Death	0	0	0	0
Stroke	0	0	0	0
Eclampsia	9 (13.0)	3 (20.0)	9 (9.2)	5 (11.4)
Hysterectomy	0	0	0	0
Placental abruption	0	3 (20.0)	1 (1.0)	0
Pulmonary oedema	0	0	0	0
Blood transfusion	3 (4.3)	2 (13.3)	7 (7.1)	4 (9.1)
Severe hypertension	60 (87.0)	13 (86.7)	68 (69.4)	31 (70.5)
Other maternal complications:	7 (10.1)	4 (26.7)	6 (6.1)	4 (9.1)
Documented primary indication for delivery by clinician (N = induced plus pre-labour CS)	n = 47	n = 12	n = 55	n = 20
Severe pre-eclampsia	34 (72.3)	9 (75.0)	40 (72.7)	15 (75.0)
Eclampsia	6 (12.8)	3 (25.0)	6 (10.9)	5 (25.0)
Other	6 (12.8)	0	9 (16.4)	0
Hospital length of stay	n = 69	n = 15	n = 98	n = 44
Median (IQR) pre-delivery length of stay (days)	1 (1–3)	1 (1–1)	1 (1–2)	1 (1–1)
Median (IQR) postnatal length of stay (days)	3 (2–5)	8 (7–11)	3 (2–4)	7 (5–9)

*Records of antenatal ultrasound or clinic visits not always available

**Table 2:** Case notes review—infant data

	34–36^+6^ weeks N (%)		≥ 37 weeks N (%)	
	Zambian sites	Indian sites	Zambian sites	Indian sites
Total number of infants (N)	n = 74	n = 16	n = 102	n = 44
Livebirths	72 (97.3)	15 (93.8)	99 (97.1)	41 (93.2)
Antepartum stillbirths	2 (2.7)	1 (6.3)	2 (2.0)	2 (4.5)
Intrapartum stillbirths	0	0	1 (1.0)	1 (2.3)
Neonatal deaths (% of livebirths)	2 (2.7)	1 (6.7)	2 (2.0)	1 (2.4)
No birth outcome reported	0	0	0	0
Mode of delivery:				
Spontaneous vaginal delivery	32 (43.2)	3 (18.75)	44 (43.1)	12 (27.2)
Assisted vaginal delivery	1 (1.4)	0	5 (4.0)	0
Caesarean section	41 (55.4)	13 (81.3)	52 (51.0)	32 (72.7)
Not documented	0	0	1 (1.0)	0
Median (IQR) gestation at delivery (days)	249 (243–252)	251 (245–255)	269 (266–280)	272 (266–282)
Median (IQR) birthweight (kg)	2.2 (1.9–2.7)	1.9 (1.8–2.3)	2.8 (2.3–3.3)	2.7 (2.5–3.0)
Median (IQR) birthweight centile*	16 (5–73)	5 (2–17)	18 (3–49)	11 (4–24)
Small for gestational age (birthweight < 10^th^ centile)	28 (38.3)	10 (62.5)	37 (36.3)	22 (50.0)
Admission to neonatal unit N (% livebirths)	37 (50.0)	13 (86.7)	32 (32.3)	17 (41.5)
Primary indication for neonatal unit admission N (% livebirths):	n = 72	n = 15	n = 99	n = 41
Prematurity	13 (18.1)	0	3 (3.0)	0
Low birthweight	3 (4.2)	3 (20.0)	1 (1.0)	1 (2.4)
Respiratory distress	3 (4.2)	5 (33.3)	1 (1.0)	4 (9.8)
Birth Asphyxia/Cyanosis	5 (6.9)	0	7 (7.1)	2 (4.9)
Jaundice	0	5 (33.3)	0	8 (19.5)
Other	0	0	1 (1.0)	2 (4.8)
No clinical indication (healthy lodger)	7 (9.7)	0	14 (14.1)	0
Not documented	6 (8.3)	0	5 (5.1)	0
Respiratory support required (and type):	9 (12.5)	5 (33.3)	5 (5.1)	8 (19.5)
Oxygen	4 (5.6)	2 (13.3)	4 (4.0)	5 (12.1)
Continuous positive airway pressure	5 (6.9)	2 (13.3)	1 (1.0)	1 (2.4)
Intubation and ventilation	0	1 (6.7)	0	2 (4.9)
Antibiotics given (and indication):	9 (12.5)	3 (20.0)	6 (6.1)	6 (14.6)
Presumed sepsis	8 (11.1)	1 (6.7)	5 (5.1)	5 (12.2)
Prematurity	1 (1.2)	0	0	0
Confirmed infection	0	2 (13.3.)	1 (1.0)	1 (2.4)
Additional clinical outcomes:				
Neonatal hypoglycaemia	0	2 (13.3)	2 (2.0)	3 (7.3)
Neonatal seizures	0	1 (6.7)	0	2 (4.9)
Nasogastric feeding required	4 (5.6)	6 (40.0)	1 (1.0)	13 (31.7)
Hypoxic ischaemic encephalopathy	0	5 (33.3)	1 (1.0)	6 (14.6)
Necrotising enterocolitis	0	0	0	0
Outcome of NICU admission N (% admissions)	n = 37	n = 13	n = 32	n = 17
Discharged alive	28 (75.7)	12 (92.3)	30 (93.8)	13 (76.5)
Died	2 (5.4)	1 (7.7)	2 (6.3)	1 (5.9)
No outcome recorded	7 (18.9)	0	0	1 (5.9)
Left against medical advice	0	0	0	2 (5.9)
Hospital length of stay				
Median (IQR) length of stay (days)	4 (2–7)	6 (1–7)	3 (2–5)	6 (4–8)

**Table 3:** Illustrative quotes

	Pregnant women	Partners	Healthcare providers
**Disease burden**			
Maternal factors			
Facilitators	In my case, this condition started with high blood pressure and swelling of body parts. It affected me so much that I was admitted to intensive care unit (ICU). This condition is related to high blood pressure *(Zambia)*	Mother may have fits, haemorrhage (*India)*	I have seen eclampsia, I have seen HELLP syndrome, I have seen pulmonary edema. I have seen stroke, I have seen a massive IC bleed three weeks back. Because of the severe pre-eclampsia we lost the mother *(Obstetrician, India)*
Barriers	Is pre-eclampsia connected to sexually transmitted diseases? *(Zambia)*	It could be, maybe you are giving her too much pressure at home that’s why that blood pressure keeps on going up *(Zambia)*	We need to sensitise them. Because mostly, you would ask the woman if at all she has heard of that condition. And she will be so surprised, asking how come it’s high, that condition, or where the BP has come from *(Midwife, Zambia)*
Infant factors			
Facilitators	I also know one woman who had high BP and got fits. Her baby died but she is fine *(India)*	I have not seen but heard about it. In fact, it happened with one of my relatives. That mother’s BP was very high and baby died inside the womb *(India)*	They could have…the baby could die whilst in utero because of the raised BP, and they could have a baby with severe asphyxia because of their condition *(Neonatal nurse, Zambia)*
Barriers	Baby will not put weight if it is born early *(India)*	Mother may have fits and stroke. Baby’s growth will be restricted because of adverse effect of high BP *(India)*	And also the risk of sepsis is also very high. Because in our set-up, if the baby is shifted to the mother’s side, her handling is more by the attendants. Improper handling. So they won’t do hand washing and things. So the risk of sepsis is very high *(Paediatrician, India)*
Health system factors			
Facilitators	This is what I can say about the dangers of high blood pressure, my sister in-law passed on due to this condition and they only managed to save the child….. So I think from this example, we can see how dangerous this condition is *(Zambia)*	I know one woman who got seizures in pregnancy due to high BP. She was admitted to hospital. Baby died but mother survived *(India)*	Quite frequently, exactly. Yeah. Almost every week we have most attention from complication from pre-eclampsia. There are those that go for severe form, they go for dialysis. They have some renal injury as well, you know (*Junior doctor, Zambia)*
Barriers	If woman has high BP then she may not understand what to do!!! (*India)*	They delayed in bringing this lady to the hospital and by the time she was brought in, the placenta had burst and the baby died in the womb as a result *(Zambia)*	Sometimes the challenge is that despite being told antenatally, these mothers who experience headaches, they remain at home until that headache is very persistent that they even fail to sleep or do anything. That’s when they come to the hospital. Sometimes it’s late, yes *(Midwife, Zambia)*
**Current management**			
Maternal factors			
Facilitators	And also maybe the swelling of the body, usually it is the legs, the hands….*(Zambia)*	I have an in-law who had high blood pressure and swelling of the body whilst she was pregnant with twins. She underwent forced labor and that’s how she was saved *(Zambia)*	First thing, I hope, first thing when they come, we give an IEC. That is heath talk. We talk to our women every day. So the health talk include danger signs in pregnancy, and what to prepare *(Midwife, Zambia)*
Barriers	Family member will decide whose life is important and who should be saved i.e. mother or baby *(India)*	Some children born early at 7th and 8th months will survive and some will not survive. My child did not survive. I feel it the destiny which decides the fate of each child. (He laughs in pain) Life and death is in the hands of god *(India)*	They are told at home no, you don’t have to agree to induction. You don’t have to agree to this. So they follow that. And they would rather follow what their parents or their relatives tell them not do it *(Midwife, Zambia)*
Infant factors			
Facilitators	At 34 weeks the baby is strong and big enough to be delivered. Overall, this will save the lives of both the mother and child. I once gave birth at 36 weeks and the baby weighed 3.8 kg *(Zambia)*	Both mother and baby will survive. Even the baby is small we can take care of baby so that it can have normal development *(India)*	I mean, as I said, between 34 and 37 weeks, babies are normal with none of these co-morbidities. Outcome will be good with monitoring *(Paediatrician, India)*
Barriers	Baby was very small so kept in the incubator. The cost of treatment was very high so could afford to keep baby in NICU for 4 days and then took the baby home against medical advice. In home they tried to take care of baby. They used Hot water bottle to keep baby warm. Baby survived for 21 or 27 days and then died *(India*)	Baby may require more care and medication. Apart from this, I do not know much (*India)*	Okay. So there are some things that I think…of course we are professional, but you may know them when you are in the shoes of the patient. So for example I think it is easy as a doctor to say give the baby medicine three times a day, but you don’t know the actual struggle that the mother goes through to make those babies swallow that medicine *(Junior doctor, Zambia)*
Health system factors			
Facilitators	So I think they want to deliver you before you get to the stage were you might start fitting and the like *(Zambia)*	I tell people who had high BP to go to hospital early and deliver early by caesarean section or else mother will die *(India)*	Gestational hypertension means only the high BP. Then pre-eclampsia means they’ll have all the categories. They have proteinuria, pedal enema, it may have abdominal wall oedema. They have them *(Community healthcare worker, India)*
Barriers	Just to add a few words, sometimes when we pregnant women go for antenatal clinics, they tell us medical terms that we can’t understand (*Zambia)*	If it is indicated to deliver it is better to deliver and if you delay in such condition people will scold you (*India)*	Because the few vents, we have like four vents on the unit. And if I have six babies, obviously two babies won’t be put on the vent, and then they actually end up dying *(Paediatrician, Zambia)*
**Acceptability**			
Maternal factors			
Facilitators	We would be able to save the life of the mother and the baby (*Zambia)*	On my own behalf, rather than losing my spouse I would say anyway, just do false labor (*Zambia)*	Okay. First of all we are going to preserve the mother’s life, we are going to prevent her from tipping into severe PE. Yeah *(Midwife, Zambia)*
Barriers	Urban people cannot tolerate labour pain so they prefer to deliver by caesarean section *(India)*	Then on the disadvantages I think it’s the actual forcing of labor before it’s due. Like everything else that’s forced, this in itself is a disadvantage. For example, in forced labor medicine is used to induce it, these medicines have side effects. God himself meant for pregnancy to last for 9 months before labor can start, but before that time you force it *(Zambia)*	So they tend not to understand the dangers of the condition that they have. So most of them request to go home, “sister, I want to be discharged” *(Midwife, Zambia)*
Infant factors			
Facilitators	Baby will have advantages. Baby will have less complications *(India)*	Delivering early is okay because by waiting, an expectant mother might die with the pregnancy or the child might die. The risks are just too many, so it’s better to deliver this person and save both lives (*Zambia)*	Actually I’m treating pre-term, I am really comfortable. Rather than severe asphyxia. You can’t do anything *(Paediatrician, India)*
Barriers	Maybe my worry is, I am not too sure if they are some conditions on developmental milestones that these children go through as a result of having been born too early *(Zambia)*	The baby might not have grown properly so it may have some problems *(India)*	So the thing is, when you deliver a baby at 34 weeks, obviously they are not yet mature. There are a few complications that the baby may suffer as a result of prematurity, for example physiological jaundice, their immunity’s not yet as strong, they may have to undergo septic screenings *(Junior doctor, Zambia)*
Health system factors			
Facilitators	The Doctor has the authority to save you because they have been trained to do so. This is why in the first place we go to them (Doctors) because if you did not want to be saved, you would not have come *(Zambia)*	Doctors are god so whatever they suggest we will agree for that *(India)*	Because there are those who start antenatal from the clinics, and the follow-up is not that very good. There are times when the BPs are high at the clinic and they don’t refer them, they refer them quite late at the hospital *(Midwife, Zambia)*
Barriers	If we have saving we will spend it if not we will ask any known person for help. If the patients are very poor they will sell their assets like Gold and bear the expenses of hospital in emergency to save mother and child (*India)*	We will borrow money from friends. If we have save money, we can use that. There are no insurance schemes right now to pay for expenses of pregnant woman (*India)*	One more challenge I would… many times the parents are not willing to keep the baby for such a long time. Because they feel that, I mean, the time spent, the amount and the revenues spent on these babies is not good *(Paediatrician, India)*

### Disease burden

#### Maternal factors

Case notes review data highlighted the serious maternal and perinatal morbidity associated with pre-eclampsia across sites in both countries (Tables 1, 2). Notably, n = 12 (14.3%) women who delivered between 34^+0^ and 36^+6^ weeks in Zambia experienced eclampsia, compared to n = 14 (9.2%) delivering at term (≥ 37 weeks). Placental abruption, acute kidney injury, and HELLP syndrome were also frequently recorded clinical outcomes. Between 34^+0^ and 36^+6^ weeks, n = 60 (87%) women in Zambia and n = 13 (86.7%) women in India developed severe hypertension, which supports the finding that approximately three quarters of women at this gestation underwent clinician-initiated delivery for severe pre-eclampsia. Complementing this quantitative data, women, partners and healthcare providers all demonstrated a clear understanding of the complications linked to pre-eclampsia and were able to share examples of their own lived experience, either as healthcare providers managing these complications or as patients (or patient relatives) experiencing the disease itself (Table 3). Whilst healthcare providers were able to provide more detailed accounts using medical terms, women and their partners could identify links between raised blood pressure and serious complications such as death, stroke and eclampsia (“fits”). However, potential barriers to understanding were also highlighted. For example, misconceptions surrounding the underlying cause of pre-eclampsia were identified, with women and partners sometimes making connections between raised blood pressure and emotional states, and healthcare providers identifying a need to improve awareness around the condition.

#### Infant factors

Overall, there were a low number of infant deaths occurring after 34 weeks’ gestation in our sample. Between 34^+0^ and 36^+6^ weeks, the proportion of antepartum stillbirths [n = 3, (3.3%)] was similar to the number of neonatal deaths [n = 3, (3.4%)]. Importantly, the proportion of neonatal deaths that occurred in infants born late preterm (34^+0^–36^+6^ weeks) and term (≥ 37 weeks) was low in both groups [n = 3, (3.3%) and n = 3, (2.1%) respectively]. Furthermore, whilst respiratory distress was a more commonly documented indication for neonatal unit admission in infants born late preterm [n = 8, (16.0%) late preterm vs. n = 5, (10.2%) term], birth asphyxia was more common in those born at term [n = 5, (10.0%) late preterm vs. n = 9, (18.4%) term]. Additionally, women, partners and healthcare providers in both countries frequently mentioned instances of infant death, with examples of the baby dying “inside the womb” the most commonly reported infant complication of pre-eclampsia. Whilst recognising this important risk associated with continuing pregnancy, healthcare providers also expressed concern regarding the risks of early delivery. Interview participants mentioned high rates of hospital-acquired infection within neonatal units, however, these concerns were not borne out by the case notes review data which demonstrated only small numbers of confirmed infection amongst infants born after 34 weeks (n = 4, 4.0% of total neonatal unit admissions). There was also a perceived concern that higher rates of growth restriction amongst infants of women with pre-eclampsia would put these infants at greater risk of complications of prematurity. However, only n = 6 (12.0%) late preterm neonatal unit admissions were due to low birthweight.

#### Health system factors

Case notes review data demonstrated that in Zambia, approximately 1 in 5 women experienced a composite outcome of severe maternal mortality or morbidity (in India, this proportion was even higher with 2 in 5 women experiencing the composite outcome, though our sample size was smaller). Healthcare providers reported witnessing complications of pre-eclampsia on a weekly if not daily basis, and women and partners were both able to recall examples of friends and family (including their own partners in the case of male participants) affected by pre-eclampsia, often with severe consequences. Thus, pre-eclampsia was perceived as an important and frequent problem by pregnant women and their partners, and healthcare providers highlighted a clear need to optimise current management. Nevertheless, potential barriers to implementing a facility-based intervention (such as planned early delivery) were identified. These centred around delayed presentations to care related in part to lack of understanding amongst the local community, as well as delayed referrals from peripheral healthcare facilities to tertiary level care.

### Current management

#### Maternal factors

Case notes review data showed that the majority of women diagnosed with pre-eclampsia met the diagnostic criteria of hypertension and proteinuria, as outlined by international guidelines [20, 20]. There was widespread use of antihypertensives and magnesium sulfate, suggesting appropriate management of those with severe disease. In accordance with World Health Organisation (WHO) guidelines on the management of pre-eclampsia, over 90% of women across both country sites were admitted to hospital once diagnosed and referred (although our predominantly urban sample based in tertiary healthcare facilities may not necessarily be generalisable to other settings). Amongst healthcare providers there was a good understanding of both diagnosis and management of pre-eclampsia and particularly the need for early delivery (Table 3). This was supported by responses from women and partners who were able to recall many of the common signs and symptoms of pre-eclampsia in addition to recognising that medical interventions (such as induction of labour) may be required in order to save a woman’s life. However, important themes identified from the focus group discussions at both Indian and Zambian sites also included a sense of fatalism and the idea that the outcome of a pregnancy would be “decided by God”, rather than medical intervention. A lack of female autonomy related to making decisions regarding healthcare was also apparent in both countries, with partners and extended family members often given the power to decide whether to proceed with an intervention such as induction of labour or caesarean section.

#### Infant factors

Neonatal outcome data collected as part of the case notes review demonstrated good neonatal outcomes between 34^+0^ and 36^+6^ weeks. Median birthweight was above 1.8 kg (the threshold for neonatal unit admission according to local protocols) in both Indian and Zambian settings. Whilst a high proportion of livebirths were admitted to the neonatal unit [n = 37, (50.0%) in Zambia, n = 13 (86.7%) in India], the majority of these infants were discharged alive [n = 28 (75.7%) in Zambia, n = 12 (92.3%) in India] and only three neonatal deaths were recorded following neonatal unit admission [n = 2 (5.4%) in Zambia, n = 1 (7.7%) in India]. The same number [n = 3 (3.4%)] of neonatal deaths were recorded for neonates born ≥ 37 weeks. Small numbers of neonates born between 34^+0^ and 36^+6^ weeks required respiratory support [n = 9, (12.5%) of neonates in Zambia and n = 5 (33.3%) of neonates in India], but serious morbidity {such as necrotising enterocolitis [n = 0] or neonatal seizures [n = 1 (2%)]} was rare at this late preterm gestation. Qualitative data complemented these findings, particularly interviews with healthcare providers who expressed confidence that after 34 weeks’ gestation, infants were likely to do well. Even amongst women and partners, there was recognition that hospitals and doctors were able to help small, premature babies and several women reported personal experiences of delivering their babies early, with positive outcomes. Nevertheless, some gaps in knowledge and understanding regarding the care of a preterm infant were identified during the focus group discussions. There was limited understanding of what a neonatal unit admission might involve and the type of support that could be provided to preterm infants, as well as examples of individuals who had attempted (sometimes unsuccessfully) to care for a preterm infant at home in order to avoid the cost of a neonatal unit admission.

#### Health system factors

Whilst maternal case notes data demonstrated robust clinical diagnosis of pre-eclampsia across the proposed trial sites and good adherence to WHO guidelines on the management of pre-eclampsia, it was also clear that resource limitations present a significant challenge in these settings. For example, only n = 5 [7.2%] women in Zambia and n = 5 [33.3%] women in India (see Additional file 3: Table 4) had an obstetric ultrasound scan before 20 weeks’ gestation, making accurate gestational age determination harder. There was a clear disparity in the availability of laboratory investigations between the two countries noted. Whilst creatinine and liver enzyme testing appeared to be routinely available at the two Indian sites, only a quarter of women in Zambia had these tests performed. No women in either country had a quantitative (e.g., protein: creatinine ratio or 24 h urinary protein collection) assessment of proteinuria performed. Whilst neonatal outcomes were reassuring, interviews with healthcare providers also highlighted a number of concerns relating to a lack of neonatal resources, in particular ventilators and medications such as surfactant and anti-convulsants. A further challenge relating to women’s willingness to accept care was identified during focus group discussions which revealed examples of poor communication between healthcare providers and women or families. These examples often related to a lack of explanation, or at times a didactic and paternalistic approach to delivering care and thus a breakdown of rapport between clinical staff and women.

### Acceptability

#### Maternal factors

When considering the perceived risks and benefits of planned early delivery from a maternal perspective, the most important perceived benefit amongst healthcare providers, women and partners was the potential to save the woman’s life and reduce the likelihood of life-threatening complications (Table 3). Whilst potential disadvantages were also identified (most notably there was a reluctance amongst women and their partners to accept early induction of labour), the benefit of preserving the woman’s life was seen to outweigh any potential risks associated with a preterm delivery. Whilst some women and partners expressed concern that induced labour may increase the need for operative delivery, this fear was not supported by case notes review data which showed that between 34^+0^ and 36^+6^ weeks, the majority of women who underwent induction of labour were able to deliver vaginally (Additional file 3: Table 4). Whilst healthcare providers expressed concerns regarding women’s willingness to accept hospital admission based on a lack of understanding of the seriousness of the condition, most women and their partners felt that they would accept medical intervention if it meant saving the life of both the woman and their baby.

#### Infant factors

The perceived risks of early delivery to the infant identified by healthcare providers, women and partners was the impact of preterm delivery and the ways in which this may affect the infant’s growth and development. However, overriding these concerns was a firm recognition of the mother-infant dyad and the idea that the best way to achieve a healthy infant was first to ensure the health of the mother. The consequences of waiting to deliver were clearly stated and included infant death due to stillbirth or severe birth asphyxia.

#### Health system factors

Considering the acceptability of planned early delivery from a health system perspective, the inherent challenges in delivering antenatal care and providing follow up for high-risk women in these settings acted as a facilitator towards the intervention as healthcare providers perceived a benefit to earlier intervention, given these challenges. Furthermore, whilst household decision making was often deferred to other family members (particularly male members of the household), women and partners demonstrated a high level of trust placed in medical professionals and ultimate decision-making authority provided to doctors. Countering this, was the perceived financial risk of a neonatal unit admission, which was highlighted as a particular issue in India, whereas care in Zambia was provided largely free of charge.

## Discussion

Assessing the disease burden due to pre-eclampsia across our study sites demonstrated the high prevalence of adverse pregnancy outcomes associated with the condition in these settings. Combining case notes data with the powerful lived experiences of healthcare providers, women and their partners highlighted a strong desire for optimising current management and confirmed a need for evaluation of our proposed intervention (planned early delivery). Whilst it is not possible to draw firm conclusions based upon our relatively small sample, the infant data suggests there is no increased risk of neonatal mortality associated with late preterm delivery compared to term delivery in this high-risk population, and that prolonging pregnancy in this situation may be at least as risky to the infant as iatrogenic preterm delivery. In particular, there appears to be a higher risk of hypoxic brain injury secondary to severe maternal disease amongst infants born at term, compared to those born late preterm. Supporting this, a surprising finding was the positive attitude of paediatric doctors towards planned early delivery. Interview data showed that despite our concern that these individuals may perceive greater risk associated with the intervention, they felt more confident in managing late prematurity as compared to birth asphyxia following an emergency delivery for severe pre-eclampsia, and therefore attributed greater benefit to planned early delivery. Overall, neonatal outcome data provided reassuring evidence that the proposed trial sites have the facilities and skills to appropriately manage late prematurity. Data from the case notes review and stakeholder interviews identified key resource limitations which influenced the design of the interventional trial protocol. In particular, we were able to modify the eligibility criteria and refine our selection of maternal and perinatal outcomes, developing pragmatic, clinical definitions that would enable these variables to be measured reliably. Important facilitators assessed as part of current management included a strong recognition of the signs and symptoms of pre-eclampsia and an understanding of the need for hospital admission and early delivery. This reflects the fact that in our study settings, there is positive engagement with antenatal care [15, 16, 16] and good provision of the WHO recommended [23] ‘Information, Education, Communication’ sessions to women during these visits. Whilst healthcare providers, women and their partners did perceive some risk associated with planned early delivery (such as undergoing induction of labour or the costs of a preterm delivery), overall the intervention was found to be acceptable to the majority of stakeholders with clear perceived benefits identified (reducing the risk of death, serious complications and stillbirth) that were felt to outweigh any potential disadvantages. Our findings therefore suggest that, with appropriate modifications to suit the local context, the interventional phase of the trial would be feasible to deliver and acceptable both to those delivering the intervention (healthcare providers) and those receiving it (pregnant women with pre-eclampsia).

The mixed-methods design of this study enabled the integration of data from multiple sources. Qualitative data was used to explore and explain quantitative findings, with case notes review data also validating (or in some cases dispelling) key themes identified in analyses of focus group discussions and interviews. Case notes review data provided important findings relating to current management of pre-eclampsia as well as the availability of specific resources and the incidence of severe morbidity. This enabled an objective assessment of feasibility, and rigorous case-finding and data collection provided a complete and realistic assessment over a three-month period. The acceptability of the intervention, and the perceived risks and benefits of planned early delivery, were assessed qualitatively and this enabled a methodical and thorough understanding of knowledge, attitudes and beliefs amongst local pregnant women and their partners. This sample of focus group participants was deliberately selected to be representative of the target study population for the main trial. Focus group data has therefore informed our recruitment strategy when designing the trial protocol and ensured engagement of local stakeholders from the outset. Our study was limited by challenges with documentation, for example, despite extensive efforts it was not always possible to locate antenatal and neonatal records and thus capture all outcomes. Additionally, further research may elucidate the role of sociodemographic influences on decision-making (e.g., around pregnancy interventions). The position of the research team facilitating focus group discussions as midwives and researchers was both a strength and a limitation. For example, as midwives they were able to build trust and rapport with colleagues and women; however, this role may also have created a power imbalance between facilitator and participants. Steps were taken to counter this, for example, acting as facilitators at healthcare facilities where they did not work clinically.

Our study findings enabled us to modify implementation of the main trial in order to suit the local context. For example, in order to address common misconceptions regarding the causes of pre-eclampsia and management of preterm birth, we developed brief educational videos to supplement trial recruitment materials. Recognising the involvement of male partners and learning from previous experiences of poor communication, discussions regarding trial participation would be encouraged to take place with both the woman and her partner present. Taking resource limitations into account, the CRADLE-4 trial inclusion criteria will utilise a broad definition of pre-eclampsia based on simple clinical parameters (hypertension and dipstick proteinuria) and gestational age determination based upon known last menstrual period (LMP) rather than first trimester ultrasound. However, the use of early (prior to 20 weeks) and late ultrasound will be encouraged, particularly when reliable data on LMP is not available. This is a pragmatic approach that would be transferable to similar settings. Furthermore, whilst it can be challenging to distinguish between growth restriction and early prematurity without accurate gestational age determination, we did not want to impose stringent criteria that could potentially exclude growth restricted fetuses (on the mistaken premise of prematurity before 34 weeks), who are in fact at the highest risk of intra-uterine death and potentially may benefit most from early delivery. Clinical outcomes were also adapted. The primary short-term maternal outcome used in the main trial will be based on the miniPIERS composite of adverse maternal outcomes [24], with the addition of severe hypertension. The miniPIERS composite had previously been selected for use in a prospective study of women with any hypertensive disorder of pregnancy in a low and middle-income setting [24]. We further modified the outcome definitions based upon our study findings. For example, we modified the definition of “blood transfusion” to include a request for transfusion even if blood products were unavailable at time of request or not received. Acknowledging the discrepancy in biochemistry testing between sites, we also plan to report a separate maternal mortality and morbidity composite of components detected by a clinical diagnosis only, as a secondary maternal outcome. Perinatal outcomes were also adapted via iterative discussion with site teams, building upon findings from stakeholder interviews with paediatric staff. For example, recognising that culture-proven sepsis is a difficult outcome to detect due to limited laboratory resources, a diagnosis of possible serious bacterial infection (based on WHO’s Integrated Management of Childhood Illness guidelines [25]) was added as a secondary perinatal outcome.

Based upon the maternal and neonatal outcome data collected during the case notes review, we anticipate a maternal event rate composite outcome of severe maternal mortality or morbidity with severe hypertension) of 80% and a neonatal event rate (stillbirth or neonatal death of neonatal unit admission for > 48 h with morbidity) of 23% in the expectant management (usual care) group of the main trial, in women with late preterm pre-eclampsia. This informed our sample size calculation, which is detailed in the published trial protocol [26].

The Medical Research Council guidelines on developing and evaluating complex interventions recognise that interventions are often undermined by problems of acceptability, compliance, delivery of the intervention, recruitment, and retention [27]. The guidelines therefore advocate that initial feasibility studies are undertaken in order to address these potential issues when designing the main study protocol. Considering an intervention such as planned early delivery in pre-eclampsia in India and Zambia, there are several behaviours required by those delivering the intervention (healthcare providers) and those receiving it (women) which are complex and need to be understood. Selecting meaningful maternal and perinatal outcomes, which can be reliably measured in a real-world setting, was also a potential challenge. Despite its importance, feasibility work is often poorly described and under-reported [7]. The CRADLE-4 feasibility study therefore serves as an important example of how the Medial Research Council Guidelines on developing and evaluating complex interventions can be put into practice and used to guide the development of a randomised trial design. Furthermore, there is currently inconsistent reporting of outcomes from randomised trials evaluating interventions for pre-eclampsia [28], leading to the potential omission of clinically important outcomes and difficulty in comparing and contrasting individual studies, thus limiting our ability to draw firm conclusions from the evidence available. Recent work has therefore focussed on the develop of a core outcome set for pre-eclampsia research [29]. The CRADLE-4 trial, informed by its feasibility phase, presents an opportunity to develop and validate these core outcomes, such that they may be shared and used in future pre-eclampsia trials taking place in similar settings.

## Conclusion

Pre-eclampsia is a progressive and unpredictable disease and deciding when to recommend delivery presents a challenging scenario to clinicians around the world. The balance of risks and benefits must be carefully weighed depending on the gestational age of the pregnancy and the severity of the condition. When considering the specific gestational window between 34^+0^ and 36^+6^ weeks, it is clear that planned early delivery is likely to reduce adverse maternal outcomes, but further clarity is needed regarding impact on neonatal outcomes and other key maternal considerations such as mode of delivery. Our preliminary findings from this study suggest that whilst planned early delivery may involve an increased risk of neonatal unit admission with small numbers of babies requiring additional support with feeding and breathing, continuing with expectant management poses a significant risk of stillbirth and birth asphyxia. A larger scale randomised controlled trial is needed to fully evaluate which management strategy poses the least risk overall. This feasibility study has demonstrated that whilst contextual challenges related to the proposed trial environment need to be taken into consideration, such a trial is indeed feasible and the proposed intervention is acceptable to local stakeholders (healthcare providers, women and their partners). These preliminary findings have directly influenced the design of the interventional phase protocol, specifically the selection of outcome measures, with a view to contributing towards core outcome sets for similar trials taking place in low- or middle-income settings. Staff training and participant recruitment materials will address the gaps in knowledge identified during focus group discussions and interviews as well as fears and fixed beliefs surrounding early delivery. Co-creating a trial protocol with local stakeholders at this stage and taking into account the feasibility and acceptability of the intervention will be key in ensuring that any evidence generated as part of this research can be successfully implemented and sustained within routine clinical practice.

## Supplementary Information

**Additional file 1.** Focus group discussion guide example (women).

**Additional file 2.** Interview topic guide.

**Additional file 3.** Tables 4, 5 Case notes review data supplementary tables.

## Data Availability

All data generated or analysed during this study are included in this published article and its supplementary information files.

## Abbreviations

LMIC: Low and middle-income countries
REDCap: Research electronic data capture tools
CRF: Case report form
NICU: Neonatal intensive care unit
HELLP syndrome: Haemolysis, elevated liver enzymes, low platelet count
WHO: World Health Organisation
LMP: Last menstrual period
