# Supplementary material for: Planned early delivery for late preterm pre-eclampsia in a low- and middle-income setting: a feasibility study
Source: Reprod Health. 2021 Jun 2;18:110. doi: 10.1186/s12978-021-01159-y (PMC8173959; doi:10.1186/s12978-021-01159-y)
Supplement: Supplementary file 1 — Additional file 1. Focus group discussion guide example (women). [file 12978_2021_1159_MOESM1_ESM.docx]

**CRADLE-4 Phase 1 Focus Group Guide**

The purpose of this focus group is:

- To explore women’s experiences and understanding of pre-eclampsia and pre-term birth through a group discussion.
- To explore women’s views surrounding planned early delivery in pre-eclampsia

Reassure the participants that their answers will be anonymous and we would like them to be honest. These questions are a guide only; feel free to explore topics as they arise.

Thank you all for agreeing to participate today.

| We’re going to start by talking about a condition called pre-eclampsia. | *What do you understand about this condition?*  *Do you know anyone who has had pre-eclampsia?* |
| --- | --- |
| What impact might pre-eclampsia have on a woman’s pregnancy and her baby? | *How might it affect the mother?*  *How might it affect the baby?* |
| Sometimes women with pre-eclampsia might have their babies early… | *What do you understand about this?*  *How do you feel about this?*  *Has this happened to anyone you know?*  *Can you tell me any more about this?* |
| If a woman with pre-eclampsia has her baby early what do you think the advantages and disadvantages might be? | *For the woman?*  *For the baby?* |
| Do you know anyone who has had a baby that was born early? | *What happened?*  *Can you tell me more about this?* |
| What care might be provided to babies who are born early? | *What care might be needed?*  *What care is available here?*  *How might this care be accessed?*  *Are there any barriers to accessing this care?*  *Can you tell me more about this?* |

Pre-eclampsia can be a serious condition for both mother and baby. It can cause very high blood pressure in the mother and in serious cases it can cause fits, strokes, organ failure and sometimes death. Babies whose mothers have pre-eclampsia tend to be smaller and are more likely to be born early. Because of this, most women with pre-eclampsia have their labour started around 37 weeks of pregnancy.

But, because some of the complications of pre-eclampsia can be life-threatening and the condition of both the mother and baby can suddenly worsen, some doctors think it may be better for women with pre-eclampsia to have their babies before this.

We are designing a trial to find out whether, in women with pre-eclampsia between 34 and 37 weeks of pregnancy, planned early delivery causes fewer complications for the mother and/or baby, compared to waiting until 37 weeks (unless a serious problem occurs before this time).

This means that women who agree to participate in the trial will be randomly allocated to planned early delivery or expectant management (watchful waiting).

| What do you think about this? | *Can you tell me why?* |
| --- | --- |
| What do you think the advantages and disadvantages of being in the early delivery group might be? | *For the mother?*  *For the baby?*  *Can you tell me more about this?* |
| What do you think the advantages and disadvantages of being in the watchful waiting group might be? | *For the mother?*  *For the baby?*  *Can you tell me more about this?* |
| Planned early birth is usually started by a process called “Induction” | *What do you understand by this?*  *Do you know anyone who has had an Induction?*  *What do you think about Induction?*  *Can you tell me more about this?* |
| Occasionally, planned early birth might happen by caesarean section (for example, if a woman has had a caesarean section in the past) | *What do you understand by this?*  *Do you know anyone who has had a caesarean section?*  *What do you think about caesarean section?*  *Can you tell me more about this?* |
| How would you prefer to give birth? | *Can you tell me why?* |
| How do you feel about planning early birth in women with pre-eclampsia? | *Can you tell me why?* |
| How does your family feel about pregnancy and childbirth? | *How might their views influence your preferences?*  *Do you have a husband? How might his views influence your preferences?* |
| How do you get to the hospital? | *At short notice?*  *Can you tell me more about this?* |
| How do you pay for your maternity care? | *Can you tell me more about this?* |
| How would you feel if you or your baby needed to spend additional time in hospital? | *Can you tell me more about this?* |
