# Supplementary material for: Planned early delivery for late preterm pre-eclampsia in a low- and middle-income setting: a feasibility study
Source: Reprod Health. 2021 Jun 2;18:110. doi: 10.1186/s12978-021-01159-y (PMC8173959; doi:10.1186/s12978-021-01159-y)
Supplement: Supplementary file 2 — Additional file 2. Interview topic guide. [file 12978_2021_1159_MOESM2_ESM.docx]

**CRADLE-4 Phase 1: Stakeholder Interview Guide**

The purpose of this interview is:

- To understand the experience of healthcare providers managing women with pre-eclampsia and their babies in this hospital.
- To explore healthcare providers’ views surrounding management of pre-eclampsia and pre-term birth
- To find out how healthcare providers feel about planned early delivery in women with pre-eclampsia.

Reassure the interviewee that their answers will be anonymous and we would like them to be honest.

| **Introductory Questions** |  |
| --- | --- |
| How long have you been working in this healthcare facility? |  |
| What is your role? |  |
| Can you tell me about your understanding of pre-eclampsia? |  |
| Do you work with women or babies that have been affected by pre-eclampsia? | *Can you tell me more about your experience of this?* |
| **Exploratory Questions** |  |
| What is your understanding of how pre-eclampsia is managed in this facility? | *At what gestation are women with pre-eclampsia routinely delivered in this hospital?*  *What do you think the advantages and disadvantages of this might be?*  *(If Obstetrician) How do you manage someone with pre-eclampsia?* |
| Can you tell me about your understanding of what happens to women who have pre-eclampsia? | *What happens to these women?*  *What is your experience of this?* |
| Can you tell me about your understanding of what happens to the babies of women who have pre-eclampsia? | *What happens to these babies?*  *What is your experience of this ?* |
| Can you tell me about your experience of the neonatal facilities here? | *What happens to babies that are born before term?*  *How are they looked after?*  *What are their outcomes?*  *How do you feel about the management of pre-term babies in this facility?* |
| How do you feel about the management of pre-eclampsia in this facility? | *What works well?*  *What do you find difficult?*  *What could be improved?* |

After 37 weeks’ gestation we know that the World Health Organisation recommends delivery in women with pre-eclampsia. But, because some complications of pre-eclampsia can be life-threatening and the condition of both the mother and baby can suddenly worsen, some clinicians think it may be better for women with pre-eclampsia to have their babies earlier than this.

We are designing a trial to find out whether, in women with pre-eclampsia between 34 and 37 weeks of pregnancy, planned early delivery causes fewer complications for the mother and/or baby, compared to waiting until 37 weeks (unless a serious problem occurs before this time).

This means that women who agree to participate in the trial will be randomly allocated to planned early delivery or expectant management (watchful waiting).

A similar trial is currently underway in the U.K. but it’s important to evaluate the intervention in other settings where the outcomes may be different.

| What do you think about this? | *What do you think the advantages and disadvantages of early delivery might be?*  *What do you think the advantages and disadvantages of expectant management might be?* |
| --- | --- |
| How do you think the care provided to women in each group might differ? | *How might the care of women in the early delivery group differ from routine management?* |
| What might the advantages and disadvantages be for the babies in each group? | *What do you think the advantages and disadvantages of early delivery might be for the baby?*  *What do you think the advantages and disadvantages of expectant management might be for the baby?* |
| What impact do you think the trial will have on the capacity of your unit? | *(If obstetrician/midwife) How might it affect labour ward?*  *(If neonatal nurse/doctor) How might it affect the neonatal unit?* |
| What do you hope the trial might achieve? | *Can you tell me more about that?* |
| Do you have any concerns or questions about the trial? | *Can you tell me more about that?* |
