# Supplementary material for: Planned early delivery for late preterm pre-eclampsia in a low- and middle-income setting: a feasibility study
Source: Reprod Health. 2021 Jun 2;18:110. doi: 10.1186/s12978-021-01159-y (PMC8173959; doi:10.1186/s12978-021-01159-y)
Supplement: Supplementary file 3 — Additional file 3. Tables 4, 5 Case notes review data supplementary tables. [file 12978_2021_1159_MOESM3_ESM.docx]

**Case notes review: Table 4 Maternal data (supplementary tables)**

|  | **<34 weeks N (%)** | | **34-36^+6^ weeks N (%)** | | **≥37 weeks N (%)** | |
| --- | --- | --- | --- | --- | --- | --- |
|  | Zambian sites | Indian sites | Zambian sites | Indian sites | Zambian sites | Indian sites |
| Total number of women | n=87 | n=13 | n=69 | n=15 | n=98 | n=44 |
| **Maternal characteristics** |  |  |  |  |  |  |
| Mean (SD) age (years) | 28.8 (7.4) | 24.2 (3.3) | 26.5 (7.0) | 24.5 (3.2) | 25.8 (5.9) | 24.4. (4.2) |
| Primiparous | 30 (34.5) | 8 (61.5) | 28 (40.5) | 10 (66.7) | 57 (58.2) | 31 (70.5) |
| Singleton pregnancy | 81 (93.1) | 13 (100) | 64 (92.8) | 14 (93.3) | 94 (95.9) | 44 (100) |
| Ultrasound scan during pregnancy | 60 (69.0) | 6 (46.1) | 44 (63.8) | 8 (53.3) | 63 (64.3) | 33 (75.0) |
| Ultrasound before 20 weeks’ gestation | 4 (4.6) | 3 (23.1) | 5 (7.2) | 5 (33.3) | 7 (7.1) | 24 (54.5) |
| **At pre-eclampsia diagnosis** |  |  |  |  |  |  |
| SBP ≥140 or DBP ≥90 mmHg | 80 (92.0) | 13 (100) | 68 (98.6) | 11 (73.3) | 93 (94.9) | 30 (68.2) |
| ≥ 1 + protein on urine dipstick | 77 (88.5) | 8 (61.5) | 62 (89.9) | 8 (53.3) | 83 (84.7) | 21 (47.7) |
| Quantitative assessment of proteinuria | 0 | 0 | 0 | 0 | 0 | 0 |
| Creatinine tested | 43 (49.4) | 13 (100) | 18 (26.1) | 15 (100) | 23 (23.5) | 42 (95.5) |
| Median (IQR) creatinine value (µmol/L) | 86 (69-105) | 65 (53-80) | 72 (63-83) | 67 (55-80) | 66 (57-97) | 62 (53-80) |
| Liver enzymes tested | 47 (54.0) | 13 (100) | 24 (34.8) | 15 (100) | 24 (24.5) | 42 (95.5) |
| Median (IQR) alanine transaminase level (U/l) | 27 (14-48) | 15 (12-18) | 12 (11-28) | 18 (13-47) | 17 (13-31) | 15 (12-20) |
| Median (IQR) aspartate aminotransferase level (U/l) | 42 (32-59) | 20 (17-30) | 30 (21-42) | 22 (15-44) | 33 (24-44) | 25 (18-30) |
| Platelets tested | 73 (83.9) | 12 (92.3) | 49 (71.0) | 15 (100) | 60 (61.2) | 41 (93.2) |
| Median (IQR) platelets level (x10^9^/l) | 169 (98-231) | 217 (167-217) | 174 (146-242) | 166 (122-262) | 190 (142-260) | 211 (181-266) |
| **Pre-eclampsia management** |  |  |  |  |  |  |
| Given antihypertensives | 78 (89.7) | 13 (100) | 61 (88.4) | 15 (100) | 88 (89.8) | 35 (79.5) |
| >1 antihypertensive agent | 72 (87.8) | 10 (76.9) | 56 (81.6) | 8 (53.3) | 70 (71.4) | 14 (31.8) |
| Received antenatal corticosteroids | 63 (72.4) | 4 (30.8) | 42 (60.9) | 4 (26.7) | 9 (9.2) | 1 (2.3) |
| Received magnesium sulfate | 77 (88.5) | 10 (76.9) | 47 (68.1) | 12 (80.0) | 61 (62.2) | 19 (43.2) |
| Admitted antenatally | 78 (89.7) | 13 (100) | 66 (95.7) | 15 (100) | 90 (91.8) | 44 (100) |
| **Onset of labour** |  |  |  |  |  |  |
| Spontaneous | 14 (16.1) | 2 (15.4) | 22 (31.9) | 3 (20.0) | 43 (43.9) | 24 (54.5) |
| Induced | 46 (52.9) | 5 (38.5) | 25 (34.8) | 4 (26.7) | 28 (28.6) | 5 (11.4) |
| Pre-labour caesarean section | 25 (28.7) | 6 (46.1) | 22 (31.9) | 8 (53.3) | 27 (27.6) | 15 (34.1) |
| Not documented | 2 (2.3) | 0 | 0 | 0 | 0 | 0 |
| **Composite of severe maternal mortality and morbidity (N women)** | 29 (33.3) | 9 (69.2) | 12 (17.4) | 5 (33.3) | 17 (17.3) | 8 (18.2) |
| **Individual components (non-exclusive events)** |  |  |  |  |  |  |
| Death | 0 | 0 | 0 | 0 | 0 | 0 |
| Stroke | 0 | 0 | 0 | 0 | 0 | 0 |
| Eclampsia | 15 (17.2) | 7 (53.8) | 9 (13.0) | 2 (13.3) | 9 (9.2) | 5 (11.4) |
| Hysterectomy | 0 | 0 | 0 | 0 | 0 | 0 |
| Placental abruption | 2 (2.3) | 0 | 0 | 3 (20.0) | 1 (1.0) | 0 |
| Pulmonary oedema | 1 (1.1) | 0 | 0 | 0 | 0 | 0 |
| Blood transfusion | 11 (12.6) | 2 (15.4) | 3 (4.3) | 2 (13.3) | 7 (7.1) | 4 (9.1) |
| **Additional clinical outcomes:** |  |  |  |  |  |  |
| Severe hypertension | 74 (85.0) | 12 (92.3) | 60 (87.0) | 13 (86.7) | 68 (69.4) | 21 (47.7) |
| Post-partum haemorrhage | 3 (3.4) | 0 | 2 (2.9) | 2 (13.3) | 4 (4.1) | 0 |
| Acute Kidney Injury | 2 (2.3) | 1 (7.7) | 0 | 0 | 0 | 0 |
| Haemolysis, elevated liver enzymes and low platelet count (HELLP) Syndrome | 8 (9.2) | 1 (7.7) | 3 (4.3) | 1 (6.6) | 1 (1.0) | 3 (6.8) |
| Haemodialysis | 1 (1.1) | 0 | 0 | 0 | 0 | 0 |
| Intensive care unit admission | 2 (2.3) | 0 | 1 (1.4) | 0 | 1 (1.0) | 1 (2.2) |
| Sepsis | 2 (2.3) | 0 | 1 (1.4) | 1 (6.6) | 0 | 0 |
| Vaginal delivery N (% induced deliveries) | 36 (78.3) | 5 (100) | 16 (64.0) | 4 (100) | 13 (46.4) | 4 (80.0) |
| **Documented primary indication for delivery by clinician (N = induced plus pre-labour CS)** | n=71 | n=11 | n=47 | n=12 | n=55 | n=20 |
| Severe pre-eclampsia | 40 (56.3) | 3 (27.2) | 34 (72.3) | 9 (75.0) | 40 (72.7) | 15 (75.0) |
| Eclampsia | 15 (21.1) | 7 (63.6) | 6 (12.8) | 3 (25.0) | 6 (10.9) | 5 (25.0) |
| **Other:** |  |  |  |  |  |  |
| Intra-uterine fetal death | 13 (18.3) | 1 (9.0) | 2 (4.3) | 0 | 0 | 0 |
| Placental abruption | 1 (1.4) | 0 | 1 (2.1) | 0 | 1 (1.8) | 0 |
| Severe hypertension | 1 (1.4) | 0 | 0 | 0 | 2 (3.6) | 0 |
| Fetal distress | 0 | 0 | 3 (6.4) | 0 | 4 (7.2) | 0 |
| Reached 37 weeks’ gestation | 0 | 0 | 0 | 0 | 1 (1.8) | 0 |
| Indication not documented | 1 | 0 | 1 | 0 | 1 (1.8) | 0 |
| **Hospital length of stay** | n=87 | n=13 | n=69 | n=15 | n=98 | n=44 |
| Median (IQR) pre-delivery length of stay (days) | 2 (1-4) | 1 (1-2) | 1 (1-3) | 1 (1-1) | 1 (1-2) | 1 (1-1) |
| Median (IQR) postnatal length of stay (days) | 4 (3-6) | 12 (9-12) | 3 (2-5) | 8 (7-11) | 3 (2-4) | 7 (5-9) |

**Case Notes Review Data: Table 5 Infant data – supplementary tables**

|  | **<34 weeks N (%)** | | **34-36^+6^ weeks N (%)** | | **≥37 weeks N (%)** | |
| --- | --- | --- | --- | --- | --- | --- |
|  | Zambian sites | Indian sites | Zambian sites | Indian sites | Zambian sites | Indian sites |
| **Number of infants (N)** | n=93 | n=13 | n=74 | n=16 | n=102 | n=44 |
| Livebirths | 56 (60.2) | 8 (61.5) | 72 (97.3) | 15 (93.8) | 99 (97.1) | 41 (93.2) |
| Antepartum stillbirths | 31 (33.3) | 3 (23.1) | 2 (2.7) | 1 (6.3) | 2 (2.0) | 2 (4.5) |
| Intrapartum stillbirths | 4 (4.3) | 2 (15.4) | 0 | 0 | 1 (1.0) | 1 (2.3) |
| Neonatal deaths (% of livebirths) | 15 (26.8) | 3 (37.5) | 2 (2.7) | 1 (6.7) | 2 (2.0) | 1 (2.4) |
| No birth outcome reported | 2 (2.2) | 0 | 0 | 0 | 0 | 0 |
| **Mode of delivery:** |  |  |  |  |  |  |
| Spontaneous vaginal delivery | 47 (50.5) | 5 (38.5) | 32 (43.2) | 3 (18.75) | 44 (43.1) | 12 (27.2) |
| Assisted vaginal delivery | 0 | 0 | 1 (1.4) | 0 | 5 (4.0) | 0 |
| Caesarean section | 43 (46.2) | 8 (61.5) | 41 (55.4) | 13 (81.3) | 52 (51.0) | 32 (72.7) |
| Not documented | 3 (3.2) | 0 | 0 | 0 | 1 (1.0) | 0 |
| **Median (IQR) gestation at delivery (days)** | 212 (196-224) | 206 (189-223) | 249 (243-252) | 251 (245-255) | 269 (266-280) | 272 (266-282) |
| **Median (IQR) birthweight (kg)** | 1.4 (1-1.7) | 1.2 (0.8-1.3) | 2.2 (1.9-2.7) | 1.9 (1.8-2.3) | 2.8 (2.3-3.3) | 2.7 (2.5-3.0) |
| Median (IQR) birthweight centile* | 23 (3-76) | 7 (3-42) | 16 (5-73) | 5 (2-17) | 18 (3-49) | 11 (4-24) |
| Small for gestational age (birthweight <10^th^ centile) | 28 (30.1) | 7 (53.8) | 28 (38.3) | 10 (62.5) | 37 (36.3) | 22 (50.0) |
| **Admission to neonatal unit N (% livebirths)** | 48 (85.7) | 8 (100) | 37 (50.0) | 13 (86.7) | 32 (32.3) | 17 (41.5) |
| **Primary indication for neonatal unit admission N (% livebirths):** | n=56 | n=8 | n=72 | n=15 | n=99 | n=41 |
| Prematurity | 37 (66.1) | 3 (37.5) | 13 (18.1) | 0 | 3 (3.0) | 0 |
| Low birthweight | 2 (3.6) | 0 | 3 (4.2) | 3 (20.0) | 1 (1.0) | 1 (2.4) |
| Respiratory distress | 4 (7.1) | 5 (62.5) | 3 (4.2) | 5 (33.3) | 1 (1.0) | 4 (9.8) |
| Birth Asphyxia/Cyanosis | 2 (3.6) | 0 | 5 (6.9) | 0 | 7 (7.1) | 2 (4.9) |
| Jaundice | 0 | 0 | 0 | 5 (33.3) | 0 | 8 (19.5) |
| Other | 0 | 0 | 0 | 0 | 1 (1.0) | 2 (4.8) |
| No clinical indication (healthy lodger) | 1 (1.8) | 0 | 7 (9.7) | 0 | 14 (14.1) | 0 |
| Not documented | 2 (3.6) | 0 | 6 (8.3) | 0 | 5 (5.1) | 0 |
| **Respiratory support required (and type):** | **16 (28.6)** | **7 (87.5)** | **9 (12.5)** | **5 (33.3)** | **5 (5.1)** | **8 (19.5)** |
| Oxygen | 5 (8.9) | 1 (12.5) | 4 (5.6) | 2 (13.3) | 4 (4.0) | 5 (12.1) |
| Continuous positive airway pressure | 8 (14.3) | 1 (12.5) | 5 (6.9) | 2 (13.3) | 1 (1.0) | 1 (2.4) |
| Intubation and ventilation | 1 (1.8) | 5 (62.5) | 0 | 1 (6.7) | 0 | 2 (4.9) |
| **Antibiotics given (and indication):** | 13 (23.2) | 7 (87.5) | 9 (12.5) | 3 (20.0) | 6 (6.1) | 6 (14.6) |
| Presumed sepsis | 11 (19.6) | 6 (75.0) | 8 (11.1) | 1 (6.7) | 5 (5.1) | 5 (12.2) |
| Prematurity | 1 (1.8) | 0 | 1 (1.2) | 0 | 0 | 0 |
| Confirmed infection | 1 (1.8) | 1 (12.5) | 0 | 2 (13.3.) | 1 (1.0) | 1 (2.4) |
| **Additional clinical outcomes:** |  |  |  |  |  |  |
| Neonatal hypoglycaemia | 4 (7.1) | 2 (25.0) | 0 | 2 (13.3) | 2 (2.0) | 3 (7.3) |
| Neonatal seizures | 0 | 1 (12.5) | 0 | 1 (6.7) | 0 | 2 (4.9) |
| Nasogastric feeding required | 9 (16.1) | 7 (87.5) | 4 (5.6) | 6 (40.0) | 1 (1.0) | 13 (31.7) |
| Hypoxic ischaemic encephalopathy | 1 (1.8) | 4 (50.0) | 0 | 5 (33.3) | 1 (1.0) | 6 (14.6) |
| Necrotising enterocolitis | 0 | 1 (12.5) | 0 | 0 | 0 | 0 |
| **Outcome of NICU admission N (% admissions)** | n=48 | n=8 | n=37 | n=13 | n=32 | n=17 |
| Discharged alive | 27 (56.2) | 3 (37.5) | 28 (75.7) | 12 (92.3) | 30 (93.8) | 13 (76.5) |
| Died | 13 (27.1) | 3 (37.5) | 2 (5.4) | 1 (7.7) | 2 (6.3) | 1 (5.9) |
| No outcome recorded | 8 (16.7) | 0 | 7 (18.9) | 0 | 0 | 1 (5.9) |
| Left against medical advice | 0 | 2 (25.0) | 0 | 0 | 0 | 2 (5.9) |
| **Hospital length of stay** |  |  |  |  |  |  |
| Median (IQR) length of stay (days) | 5 (2-6) | 17 (8-24) | 4 (2-7) | 6 (1-7) | 3 (2-5) | 6 (4-8) |

|  | |  |  |
| --- | --- | --- | --- |
|  | |  |  |
|  | |  |  |
|  |  |  |  |
|  |  |  |  |
|  |  |  |  |
